# Genetic variations related to inflammation in suicidal ideation and behavior: A systematic review

**DOI:** 10.3389/fpsyt.2022.1003034

**Published:** 2022-10-17

**Authors:** Rabah Tamimou, Serge Lumbroso, Kevin Mouzat, Jorge Lopez-Castroman

**Affiliations:** ^1^Department of Psychiatry, Nimes University Hospital, Nimes, France; ^2^Laboratory of Biochemistry and Molecular Biology, Nimes University Hospital, University of Montpellier, Nimes, France; ^3^Institut de Génomique Fonctionnelle, University of Montpellier, CNRS-INSERM, Montpellier, France; ^4^Centro de Investigación Biomédica en Red de Salud Mental, Madrid, Spain

**Keywords:** self-injurious behavior, suicide, polymorphism, SNP, cytokines, chemokines

## Abstract

**Background/objectives:**

Immune-inflammatory changes have been found in all types of suicidal ideation and behavior (SIB), independently of associated mental disorders. Since several Single Nucleotide Polymorphisms (SNPs) affect the function of inflammation-related genes, we searched the literature for genetic variations potentially altering inflammatory processes in SIB.

**Methods:**

We included studies that looked for associations between SIB and SNPs in genes related to inflammatory processes. Case reports, literature reviews, and animal studies were excluded. Articles were retrieved from PubMed and PsycINFO databases, Google Scholar and GreySource Index until September 17th, 2022. Quality was assessed using Q-Genie.

**Results:**

We analyzed 32 studies. SIB has been associated with eighteen SNPs located in genes encoding for interleukin-8 (rs4073), C-reactive protein (rs1130864), tumor necrosis factor α (rs1800629, rs361525, and rs1099724), tumor necrosis factor receptor 2 (rs1061622), transforming growth factor β-1 (rs1982073), acid phosphatase 1 (rs7419262, rs300774), interleukin-10 (rs1800896), interferon γ (rs2430561), amino-carboxy muconate semialdehyde decarboxylase (rs2121337), interleukin 7 (rs10448044, rs10448042), macrophage migration inhibitory factor (rs755622), interleukin 1-α (rs1800587), and interleukin 1-β (rs1143634 and rs16944. A genome-wide association study reported one association at the threshold of significance with the rs300774 SNP, located in the 2p25 region containing *ACP1* gene.

**Discussion:**

The studies included were methodologically and clinically diverse and of moderate quality. Their findings suggest that some inflammation-related SNPs could increase the likelihood of SIB but the evidence to date is insufficient. Further research using gene-gene (GxG) and gene-environment (GxE) approaches is warranted.

**Systematic review registration:**

[https://www.crd.york.ac.uk], identifier [CRD42022296310].

## Introduction

Suicidal ideation and behavior (SIB) is a global phenomenon and a major public health problem. A conservative estimate suggests that at least 700,000 people die by suicide annually (1). Despite increasing research in recent years, the pathophysiological mechanisms leading to SIB remain poorly understood due to the complex interactions between multiple risk factors at different levels. Most studies are based on a stress-diathesis model involving distal-vulnerability factors (such as a family history of suicide, early-life adversity, and genetics), and proximal-stressful factors (such as psychiatric disorders, hopelessness, or acute substance abuse) (2).

Biological factors for SIB are generally studied as a distal vulnerability that increases the risk of attempting suicide under stressful circumstances (3). Indeed, SIB could be considered a clinical entity associated with a biologically impaired response to stress in three major systems: the hypothalamic pituitary adrenal axis (4), the serotoninergic system (5), and the immune-inflammatory system (6). Although more recent, the evidence linking the immune-inflammatory system with SIB is already compelling. Changes in the inflammatory response appear in many psychiatric disorders such as major depression (7), bipolar disorder (BD) (8) or schizophrenia (9), as well as in SIB (10). The extent to which inflammatory changes are specifically related to SIB beyond other psychiatric conditions is yet unclear (11).

The first biological measurement of inflammation associated with SIB was an increased concentration of soluble interleukin 2 receptors (IL-2R) in the plasma of suicide attempters (SAs) compared to healthy controls (12). An imbalance between the population of type 1 and type 2 T-helper cells and their corresponding cytokines was also observed in the blood of depressed subjects and those with suicidal ideation (SI) [Mendlovic et al. (13)]. But it was not until the last decades that evidence of a dysregulated immune system in SIB began to build up. Kim et al. (14) showed that interleukin 2 (IL-2) concentration in the blood of depressed SAs was lower than in depressed subjects who had never attempted suicide and healthy subjects. Indeed, most published studies associate SIB with changes in the levels of inflammatory-related molecules, but there are some exceptions. Interleukin 6 (Il-6) levels in the blood or cerebrospinal fluid did not differ between SAs and controls (15, 16). Plasma interleukin 4 (IL-4) levels were similar in suicide ideators compared to a control group (17) and suicide completers (SCs) compared to other SAs that did not die by suicide (15). Other studies have also failed to find significant associations between SIB and tumor necrosis factor α (TNFα) (18, 19) or interleukin 1-beta (IL-1β) (20).

Despite these conflicting findings, two meta-analyses and a systematic review confirm that SIB is associated with changes in the levels of proteins and cytokines that have pro-inflammatory proprieties such as C-reactive protein (CRP), interferon γ (IFNγ), TNFα, IL-1β, IL-6, and transforming growth factor β1 (TGF-β1), as well as anti-inflammatory IL-4 (10, 21, 22). Accordingly, the neutrophil-to-lymphocyte ratio (NLR), an inflammatory marker, is higher in SAs compared to non-attempters (23) and an excess of activated microglia, the primary immune cells of the central nervous system, is found in the brains of SCs, independently of psychiatric diagnoses (24).

Some studies have analyzed the gene expression profile in recent years. They report abnormalities of gene expression related to inflammation mostly in postmortem brain tissues but also in isolated blood monocytes of subjects with SIB. The messenger ribonucleic acid (mRNA) expression of IL-4 and interleukin 13 (IL-13) was elevated in the orbitofrontal area of suicide decedents compared with controls who died from other causes (25). Pandey et al. (26) compared depressed suicide victims with non-psychiatric controls in a cross-sectional case-control study. They found that mRNA and protein levels of pro-inflammatory cytokines (e.g., TNF-α IL-6, IL-1β, lymphotoxin A) were significantly increased and those of the anti-inflammatory cytokine interleukin 10 (IL-10) were reduced in the prefrontal cortex of suicide decedents (26). These findings are consistent with those reported by Schiweck et al. (27) using monocyte mRNA analyses of 32 inflammation-related genes. They found an association between suicide risk in major depressive disorder (MDD) and the upregulation of *IL1*α, *IL1*β, and *IL-6* gene expression in monocytes (27). More recently, mRNA expression profile analysis of various chemokines showed significant downregulation of CXCL1 (CXC chemokine ligand 1), CXCL2, CXCL3, and CCL2 in the prefrontal cortex of depressed subjects compared to non-psychiatric controls (28).

All these observations suggest a deregulation in gene expression and protein production of several inflammatory-related molecules in people with SIB, but this deregulation still remains poorly understood. This article reviews studies examining the relationship between single nucleotide polymorphisms (SNPs) in inflammation-related genes and SIB. A better knowledge of the role of these genes could clarify the mechanisms linking inflammatory abnormalities with SIB.

## Methods

The protocol for this systematic review is registered on PROSPERO (CRD42022296310).

### Eligibility criteria

Eligible studies were included using the following inclusion criteria: (1) participants presented SIB: namely suicidal ideation (SI) or suicidal behavior (SB) (attempt, suicide); (2) the study examined the association between SIB and SNPs within genes coding for proteins directly implicated in inflammation [association studies or genome-wide association studies (GWAS)]. The exclusion criteria were: (1) case reports, reviews of the literature and commentaries; (2) studies using animal models.

### Article search and sources

The search terms used for database searching were “suicide,” “inflammation” and “genetic,” using Medical Subject Headings (MeSH) terms with no time limit until 1st June, 2022 (see Supplementary material for exact search terms). Documents were sought in two databases (PubMed, PsycINFO) and in the Google Scholar and GreySource Index search engines. We also used a backward and forward snowball searching strategy by exploring the reference lists as well as the citations of the selected papers to identify additional studies.

### Study selection and data collection

Two authors (RT and JL-C) independently screened and selected the studies. In case of disagreement, they searched for consensus through discussion. Ruling by a third party was not necessary.

From the total list of papers, duplicates were first eliminated and then the titles and summaries of the articles were read to also eliminate those not fulfilling the eligibility criteria. The full text of articles meeting eligibility criteria was read. Two authors (RT and JL-C) independently extracted the data from the selected articles using data collection forms. The following information was gathered from the studies when available: polymorphisms investigated, main outcomes, genotype and allelic frequencies, haplotype, age, sex, name of the first author, publication year, location or/and ethnicity, number of cases and controls, and population type.

### Risk of bias assessment

Risk of bias was assessed using the Q-Genie tool (29). The Q-Genie Tool contains 11 items rated on a scale of 1–7 that assess the following domains: (1) Rationale for study; (2) Selection and definition of the outcome of interest; (3) Selection and comparability of comparison groups; (4) Technical classification of the exposure; (5) Non-technical classification of the exposure; (6) Other sources of bias; (7) Sample size and power; (8) *A priori* planning of analyses; (9) Statistical methods and control for confounding; (10) Testing of assumptions and inferences for genetic analyses; and (11) Appropriateness of inferences drawn from results. Two authors (RT and JL-C) independently assessed studies. An overall score was given to classify studies as poor, moderate, or good quality.

## Results

The results are presented according to the affected proteins: pro- and anti-inflammatory cytokines, CRP, and kynurenine pathway enzymes. All presented results are statistically significant unless specified otherwise. The following genes were studied: *TNF*α, tumor necrosis factor receptor 2 (*TNF-RII*), *IL-6*, *IL-8*, interleukin 8 receptor-alpha (*IL8RA*), *IFN*γ, interleukin 18 (*IL-18*), monocyte chemoattractant protein 1 (*MCP-1*), macrophage migration inhibitory factor (*MIF*), *IL-10*, interleukin 1-α (*IL-1*α), *IL-1*β, interleukin 7 (*IL-7*), *TGF-*β*1*, *CRP*, aminocarboxy muconate semialdehyde decarboxylase (*ACMSD*), hydroxyanthranilate 3,4-dioxygenase (*HAAO*), interleukin 28 Receptor-Alpha (*IL28RA*), acid phosphatase 1 (*ACP1*), cluster of differentiation (*CD44*), ADAM metallopeptidase with thrombospondin type 1 motif 14 (*ADAMTS14*), and proteasome activator complex subunit 2 (*PSME2*). The most frequently mentioned genes were *TNF*α (9 studies) followed by *ACP1* (4), *IL-1*β (3), *IL-8* (2), *IFN*γ (2), *MIF* (2), *IL-10* (2), *IL-1*α (2), *IL-7* (2), *TGF-*β*1* (2). All other genes were mentioned only in a single study.

**FIGURE 1:**
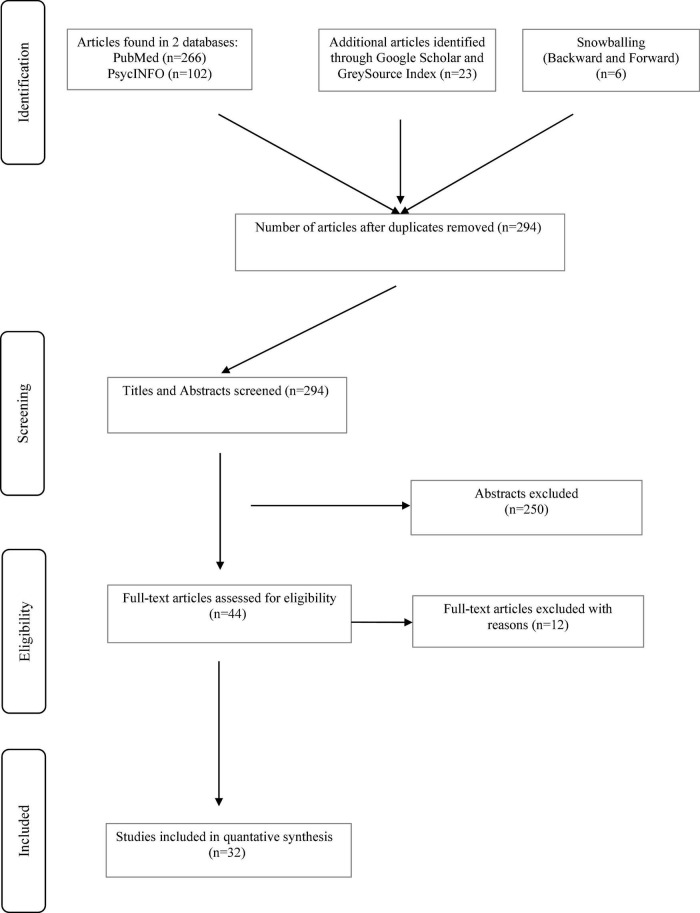
Flow chart showing study selection for the review.

Initially, 397 studies were identified. After duplicates were excluded, the remaining 294 abstracts were read and 44 were selected for full text retrieval. Twelve full-text articles were excluded (reviews, genes not directly involved in inflammation, not association studies). In total, 32 studies met the inclusion criteria (Figure 1).

The studies have been conducted in different regions of the world. USA (*n* = 7), Iran (*n* = 4), Sweden (*n* = 3), Spain (*n* = 2), Korea (*n* = 4), China (*n* = 3), Turkey (2), Brazil (2), Canada, Italy, Japan, India, and Poland. Five studies were GWAS, and all others were case-control studies (Table 1). The quality assessment classified only six studies as having good quality, fifteen with moderate quality, and eleven with poor quality (Supplementary Table 1).

**TABLE 1:** Description of the studies included in the review.

Study	Design	Gene studied	Genetic variants	Ethnic origin, location	Population type and sample size (*n*)	Age (years ± SD)	Men/Women (%)
(35)	Case-control study	*IL-1*α	rs1800587 (–889 C/T)	Caucasian, Spain	Suicide attempters: 193 Healthy controls: 420	35.60 ± 12.50 40.06 ± 11.30	36.30/63.70 51.40/48.60
		*IL-1*β	rs1143634 (+3953 C/T)				
		*TNF*α	rs1800629 (–308 A/G)				
(110)	GWAS	*IL28RA*	rs10903034	USA	Depressed with TESI: 90 Depressed without TESI: 90	NA NA	NA, matched for sex NA, matched for sex
(36)	Case-control study	*TNF*α	rs1800629 (–308 G/A)	Azari ethnicity, Northwest Iran	Suicide attempters and completers: 145 Healthy controls: 160	25.82 ± 12.57 70.6 ± 10	51/49 NA
		*IL-10*	rs1800896 (–1082 A/G)				
		*IFN*γ	rs2430561 (+874 A/T)				
(69)	Case-control study	*MCP-1*	rs1024611 (–2518 A/G)	Caucasian, Italy	Patients with mood-disorders [bipolar disorder type I (36) or II (25) or major depressive disorder (35)]: 96 Healthy controls: 161	52.41 ± 12.88 NA, matched for age	35.40/64.60 NA, matched for sex
(85)	Case-control study	*TGF-*β*1*	rs1982073 (rs1800470) (+869 T/C) rs1800471 (+915 G/C)	Korea	Depressed suicidal: 124 Depressed non-suicidal: 61 Healthy controls: 125	39.50 ± 14.50 40.10 ± 11.20 36.00 ± 09.50	33.87/66.13 34.42/65.58 43.20/56.80
(60)	Case-control study	*HAAO*	rs3755541 rs2374442 rs737148 rs3816182 rs13027051 rs3816184	Caucasian and African American	AUD with conduct disorder or suicide attempts: 511 Controls: 560	39.41 NA	70.80/29.20 NA
		*IL8RA*	rs16858808 rs16858816 rs16858811				
(77)	Case-control study	*IL-1*α *IL-1*β	rs1800587 (IL-1α -889 C/T) rs1143634 (IL-1β + 3953 C/T)	Caucasian, Spain	Suicide attempters: 227 Non-suicidal psychiatric patients: 686 Healthy controls: 420	36.30 ± 12.90 41.50 ± 12.80 40.6 ± 11.3	37/63 59.50/40.50 51.4/48.6
(63)	Case-control study	*IL-18*	rs187238 (–137 G/C) rs1946518 (–607 C/A)	China	Subjects with schizophrenia: 372 Healthy controls: 353	28.74 ± 12.58 37.01 ± 12.57	46.50/53.50 49/51
(90)	GWAS	*ACP1*	rs300774	Caucasian, Germany and USA	Bipolar suicide attempters: 1201 Bipolar patients with no history of suicide attempts: 1497	NA NA	35.14/64.86 47.97/52.03
(86)	Case-control study	*TGF-*β*1*	rs1982073 (rs1800470) (+869 T/C)	Iran	Suicide attempters: 145 Healthy controls: 200	NA NA	NA NA
(37)	Case-control study	*TNF*α	rs1800629 (–308 G/A)	Korea	Depressed suicide attempters: 204 Depressed subjects with no history of suicide: 97	39.36 ± 15.88 43.16 ± 15.43	32/68 30/70
		*IL-10*	rs1800896 (–1082 A/G)				
		*IFN*γ	rs2430561 (+874 T/A)				
(95)	Case-control study	*CRP*	rs1130864 (+1444 C/T)	Sweden	Suicide attempters: Cohort 1: 42 Cohort 2: 64 Healthy controls: 517	51.0 ± 10.1 38 ± 14.01 51 (men), 42 (women)	50/50 40/60 47/53
(112)	GWAS	*CD44*	rs1467558	Caucasian	Suicide with mood disorder: 45 Suicide without mood disorder: 23 Sudden death: 31	37 ± 14 48 ± 21 53 ± 18	50/50 83/17 83/17
(54)	Case-control study	*IL-8*	rs4073 (–251 A/T)	Sweden and USA	Suicide attempters: 109 Healthy controls: 517	43.61 ± 13.88 NA	50.45/49.55 47/53
(114)	GWAS	*IL-7*	rs10448042 rs10448044	Caucasian/Canada and United Kingdom	Three independent bipolar disorder samples: 189 308 462	33.27 ± 9.48 43.06 ± 12.41 47.84 ± 11.29	63/37 40/60 33/67
(113)	GWAS	ADAMTS14 PSME2	rs6480463 rs4575	Caucasian/Germany, Canada and USA	Suicide attempters or completers 577: Subjects without SB: 1233	NA NA	925/885 NA
(105)	Case-control study	*ACMSD*	rs2121337 (T/C)	Sweden	Suicide attempters: 77 Healthy controls: 150	NA NA	NA NA
(91)	Case-control study	*ACP1*	rs300774	Caucasian	Suicide attempters with MDD or BD: 277 Healthy controls: 847	NA 37.30 ± 12	NA 48.76/52. 24
(38)	Case-control study	*TNF*α	rs1800629 (–308 G/A)	Arapiraca (Brazil)	Mental disorders and a history of suicide attempts: 161 Mental disorders and no history of suicide attempts: 145 Healthy controls: 175	37.9 ± 11.3 40.7 ± 13.5 36.8 ± 13.1	19/81 13/87 12/88
		*IL-10*	rs1800871 (–819 C/T)				
(93)	Case-control study	*ACP1*	rs4447635 rs7419262	Caucasian	Bipolar disorders subjects: 303 Healthy controls: 238	42.97 ± 12.95 45.68 ± 20.00	29.70/70.30 34.45/65.55
(72)	Case-control study	*MIF*	rs755622 (–173 G/C) rs5844572 (–794CATT_5–8_)	Japan	Suicide completers: 602 Healthy controls: 728	50.90 ± 17.90 54.00 ± 18.50	67.77/32.23 46.70/53.30
(92)	Case-control study	*ACP1*	rs300774	Caucasian	Schizophrenia or schizoaffective subjects: with suicide attempts: 74 without suicide attempts: 88	37.02 ± 10.26 35.03 ± 11.34	58.11/41.89 72.73/27.27
Kang et al., (39, 40)	Case-control study	*TNF*α	rs1800629 (–308 G/A) rs1799724 (–850 C/T)	South Korea	Acute coronary syndrome subjects: with suicidal ideation: 195 without suicidal ideation: 774	58.90 ± 10.60 58.00 ± 11.30	65.10/34.90 74/26
		*IL-1*β	rs16944 (–511 C/T) rs1143634 (+3953 C/T)				
(41)	Case-control study	*TNF*α	rs361525 (–238 G/A) rs1800629 (–308G/A) rs1799964 (–1031 T/C)	Quebec (Canada) and Maryland (USA)	Suicide completers: 60 Healthy controls: 35	NA NA	NA NA
(55)	Case-control study	*IL-8*	rs4073 (–251 A/T) rs2227306 (+781 C/T) rs1126647 (+2767 A/T)	Iran	Non-violent suicide attempts: 229 Suicide completers: 235 Healthy controls: 290	36.2 ± 1.3 37.01 ± 0.53 NA, matched for age	66/34 67/33 NA, matched for sex
(50)	Case-control study	*IL-6*	rs2069845 (3329 G/A) rs1800795 (–174 C/G)	Iran	Suicide attempters: 320 Suicide completers: 236 Healthy controls: 341	37.60 ± 1.40 37.01 ± 0.53 39.4 ± 2.60	66.56/33.43 66.94/33.05 68.03/31.97
(43)	Case-control study	*TNF*α	rs1800629 (–308G/A) rs1799964 (–1031 C/T)	China	Schizophrenia with SIB (suicide attempts and SI): 152 Schizophrenia without SIB: 805	44.8 ± 10.6 48.4 ± 10.1	84.21/15.79 81.36/18.64
(73)	Case-control study	*MIF*	rs755622 (–173 G/C)	Turkey	Bipolar disorder with history of suicide attempts: 30 Bipolar disorder without history of suicide attempts: 70 Healthy controls: 100	41.45 ± 11.54 NA	40/60 NA
(46)	Case-control study	*TNF-RII*	rs1061622 (+676 T/G)	Chinese population	Subject with suicidal ideation: 241 Subjects without suicidal ideation: 441	16.80 ± 0.59 16.90 ± 0.59	29.88/70.12 50.79/49.21
(79)	Case-control study	*IL-7*	rs10448044	Idu-Mishmi/Northeast India	Depressed with or without suicide attempts: 210 Healthy controls: 249	<19 years (40%), >19 years (61%) <19 years (54%) >19.0 years: 45.79%	48.10/51.90 56.22/43.78
(44)	Case-control study	*TNF*α	rs1800629 (–308G/A) rs361525 (–238 G/A)	Turkey	Schizophrenia with suicide attempts: 32 Schizophrenia without suicide attempts: 81	NA NA	NA NA

NA, unavailable; GWAS, genome-wide association study; SB, suicidal behavior; SIB, suicidal ideation and behavior; SI, suicidal ideation; AUD, alcohol use disorder; TESI, treatment-emergent suicidal ideation.

The associations between SIB and SNPs are shown in (Table 2).

**TABLE 2:** Association between suicidal behavior and SNPs in genes encoding molecules involved in inflammation.

Gene studied	Genetic variants	MAFs	Population type and sample size (*n*)	Main outcomes	Study
*TNF*α	rs1800629 (–308 G/A)	0.16	Suicide attempters: 193 Healthy controls: 420	No association was found.	(35)
	rs1800629 (–308 G/A)	0.49	Suicide attempters and completers: 145 Healthy controls: 160	The GG genotype of rs1800629 was associated with SB only in men.	(36)
	rs1800629 (–308 G/A)	0.10	Depressed suicide attempters: 204 Depressed subjects with no history of suicide: 97	The GG genotype of rs1800629 was associated with risk for suicide.	(37)
	rs1800629 (–308 G/A)	0.13	Mental disorders and a history of suicide attempts: 161 Mental disorders and no history of suicide attempts: 145 Healthy controls: 175	The rs1800629 polymorphism was associated with the number of suicide attempt.	de Medeiros Alves et al. (38)
	rs1800629 (–308 G/A)	0.10	Acute coronary syndrome subjects: with suicidal ideation: 195 without suicidal ideation: 774	The –308 A allele was associated with SI only within 2 weeks after ACS. The association did not remain significant after statistical adjustment.	(39)
	rs1800629 (–308 G/A) rs1799724 (–850 C/T)	0.10 0.14	Acute coronary syndrome subjects: with suicidal ideation: 195 without suicidal ideation: 774	SI was associated with high frequency of –850 (C/T + T/T) genotype after adjustment but only within 2 weeks of the ACS. A significant interaction of –308 G/A and –850 C/T polymorphisms on SI was found at 1 year after ACS.	(40)
	rs361525 (–238 G/A) rs1800629 (–308G/A) rs3093664 (–1031 T/C)	NA 0.16 0.25	Suicide completers: 60 Healthy controls: 35	No association was found.	(41)
	rs1800629 (–308G/A) rs1799964 (–1031 C/T)	0.055 0.18	Schizophrenia with SIB (suicide attempts and SI): 152 Schizophrenia without SIB: 805	No association was found.	(43)
	rs1800629 (–308G/A) rs361525 (–238 G/A)	0.09 0.034	Schizophrenia with suicide attempts: 32 Schizophrenia without suicide attempts: 81	The distribution of rs361525 genotype was significantly different in suicide attempters compared to non-attempters.	(44)
*TNF-RII*	rs1061622 (T > G)	0.15	Subject with suicidal ideation: 241 Subjects without suicidal ideation: 441	Among TT genotype or G allele carriers, female students were more likely to report SI compared to male students.	(46)
*IL-6*	rs2069845 (G/A) rs1800795 (–174 C/G)	0.28 0.43	Suicide attempters: 320 Suicide completers: 236 Healthy controls: 341	The rs1800795 was associated with lethality of suicide attempts. Haplotype analyses revealed an association between certain haplotype blocks and the lethality of suicide attempt.	(50)
*IL-8*	rs4073 (–251 A/T)	0.48	Suicide attempters: 109 Healthy controls: 517	The T allele of rs4073 was more frequent in women who attempted suicide than in women in the control group.	(54)
	rs4073 (–251 A/T) rs2227306 (+781 C/T) rs1126647 (+2767 A/T)	0.44 0.40 0.36	Non-violent suicide attempts: 229 Suicide completers: 235 Healthy controls: 290	The T-allele frequency of rs4073 polymorphism was significantly associated with suicide attempts. Haplotype analyses revealed a significant difference in haplotype frequencies between the three studied groups.	(55)
*IL8RA*	rs16858808 rs16858816 rs16858811	NA	AUD with conduct disorder or suicide attempts: 511 Controls: 560	All IL8RA SNPs were significantly associated with AUD, with CD and/or suicide attempt.	(60)
*IFN*γ	rs2430561 (+874A/T)	0.42	Suicide attempters and completers: 145 Healthy controls: 160	The A/A genotype was frequently associated with SB but only in men.	(36)
	rs2430561 (+874 A/T)	NA	Depressed suicide attempters: 204 Depressed subjects with no history of suicide: 97	No association was found.	(37)
*IL-18*	rs187238 (–137 G/C) rs1946518 (–607 C/A)	0.08 0.45	Subjects with schizophrenia: 372 Healthy controls: 353	The GC genotype frequency of rs187238 was significantly different between patients with aggressive actions and healthy controls.	(63)
*MCP-1*	rs1024611 (–2518 A/G)	0.26	Patients with mood-disorders [bipolar disorder type I (36) or II (25) or major depressive disorder (35)]: 96 Healthy controls: 161	No allelic and genotypic association with SB (presence/absence of suicide attempt). A higher number of suicide attempts were found in AA carriers independently of diagnosis.	(69)
*MIF*	rs755622 (–173 G/C) rs5844572 (–794CATT_5–8_)	0.21 -	Suicide completers: 602 Healthy controls: 728	No association was found.	(72)
	rs755622 (–173 G/C)	0.18	Bipolar disorder with history of suicide attempts: 30 Bipolar disorder without history of suicide attempts: 70 Healthy controls: 100	The allelic and genotypic frequencies were significantly different between. BD SAs, BD non-SAs and healthy controls.	(73)
*IL-10*	rs1800896 (–1082 A/G)	0.50	Suicide attempters and completers: 145 Healthy controls: 160	The AA genotype was more frequent in subjects with SB (SCs or SAs) compared to healthy controls.	(36)
	rs1800896 (–1082 A/G)	NA	Depressed suicide attempters: 204 Depressed subjects with no history of suicide: 97	No association was found.	(37)
*IL-1 family*	rs1800587 (IL-1α -889 C/T) rs1143634 (IL-1β + 3953 C/T)	0.29 0.26	Suicide attempters: 193 Healthy controls: 420	No association was found.	(35)
	rs1800587 (IL-1α -889 C/T) rs1143634 (IL-1β + 3953 C/T)	0.29 0.26	Suicide attempters: 227 Non-suicidal psychiatric patients: 686 Healthy controls: 420	The genotypes of IL-1α -889C/T were different between impulsive and planned suicide attempts.	(77)
	rs16944 (IL-1β -511 C/T) rs1143634 (IL-1β + 3953 C/T)	0.43 0.05	Acute coronary syndrome subjects: with suicidal ideation: 195 without suicidal ideation: 774	The –511T and + 3953T alleles were associated with SI only within 2 weeks after ACS. The association did not remain significant after Bonferroni correction.	(39)
*IL-7*	8q12-q21 region rs10448042 rs10448044	NA	Three independent bipolar disorder BD samples: 189 308 462	The 8q12-q21 (rs10448042 and rs10448044) region localized at 400 kb upstream of the IL-7 gene showed suggestive associations with suicide attempts.	(114)
	rs10448044	0.42	Depressed with or without suicide attempts: 210 Healthy controls: 249	The rs10448044 polymorphism was associated with SB in the recessive model.	(79)
*TGF-*β *1*	rs1982073 (rs1800470) (codon 10) rs1800471 (codon 25)	0.44 NA	Depressed suicidal: 124 Depressed non-suicidal: 61 Healthy controls: 125	No association was found.	(85)
	rs1982073	46.25	Suicide attempters: 145 Healthy controls: 200	The TT genotype was associated with suicide attempts.	(86)
*CRP*	rs1130864 (+1444 C/T)	0.31	Suicide attempters: Cohort 1: 42 Cohort 2: 64 Healthy controls: 517	Association between the T allele and increased risk for suicide.	(95)
*ACMSD*	rs2121337 (T/C)	0.14	Suicide attempters: 77 Healthy controls: 150	The C allele was associated with suicide attempt and increased CSF QUIN.	(105)
*HAAO*	rs3755541 rs2374442 rs737148 rs3816182 rs13027051 rs3816184	NA	AUD with conduct disorder or suicide attempts: 511 Controls: 560	All SNPs were significantly associated with the combined AUD with CD or SA phenotype.	(60)
*IL28RA*	rs10903034	NA	Depressed subjects with Treatment-Emergent SI: 90 Subjects without Treatment-Emergent SI: 90	The rs10903034 SNP of IL28RA gene showed suggestive association with TESI.	(110)
*ACP1*	2p25 region rs300774	0.18	Bipolar disorder suicide attempters:1201 Bipolar disorder with no history of suicide attempts: 1497	A GWS association was found between rs300774 and suicide attempts.	(90)
	rs4447635 rs7419262	NA	Bipolar disorder: 303 Healthy controls: 238	Significant associations were found between allelic and genotypic frequencies of rs7419262 and suicide attempt type (violent/non-violent).	(93)
	rs300774	0.13	Schizophrenia or schizoaffective subjects: with suicide attempts: 74 without suicide attempts: 88	The SNP was associated with suicide attempt in males.	(92)
	rs300774	NA	Suicide attempters with major depression or bipolar disorder: 277 Healthy controls: 847	The SNP was associated with suicide attempt in BD subjects and total group (BD + MDD) after statistical adjustment.	(91)
*CD44*	rs1467558	0.26	Suicide with mood disorder: 45 Suicide without mood disorder: 23 Sudden death: 31	The SNP showed suggestive association with completed suicide.	(112)
*ADAMTS14* and *PSME2*	rs6480463 rs4575	NA	Suicide attempters or completers 577: Subjects without SB: 1233	No GWS SNPs were detected. At the suggestive level, rs6480463 (ADAMTS14) and rs4575 (PSME2) were associated with suicide attempt or completed suicide.	(113)

SIB, suicidal ideation and behavior; SB, suicidal behavior; SI, suicidal ideation; SA, suicide attempter; SC, suicide completer; BD, bipolar disorder; ACS, acute coronary syndrome; AUD, alcohol use disorder; CD, conduct disorder; SNP, single nucleotide polymorphism; TESI, treatment-emergent suicidal ideation; GWS, genome-wide significant; CSF, cerebrospinal fluid; QUIN, quinolinic acid; MAF, minor allele frequency in controls; NA, unavailable.

### Pro- and anti-inflammatory cytokines

#### TNFα

TNFα is a pro-inflammatory cytokine (30). First discovered in 1975 (31), it belongs to the superfamily of TNFs comprising 19 members (32). It is mainly produced by macrophages (33), but also by a large range of cell types such as natural killer cells (NK), T and B cell lymphocytes (34).

The first association study compared SAs to healthy controls. No significant differences were observed in genotype or allelic frequencies of rs1800629 (–308 G/A) polymorphism (35). Omrani et al. (36) then compared SCs or SAs to healthy controls. They found that the frequency of the GG genotype of rs1800629 polymorphism was higher in those with SB. However, this result was only significant for men. In an independent sample of patients with MDD (major depressive disorder), SAs were also more likely to present the GG genotype compared to non-attempters (37). Another study compared mental-disordered patients with and without a history of SAs to healthy controls. They reported that the G allele was more frequent among SAs having made a single attempt compared to controls (38).

Kang et al. (39, 40) conducted two studies on the same cohort to investigate the relationship of SI to TNFα polymorphisms 2 weeks and 1 year after an acute coronary syndrome (ACS). In the first study, a significant association between SI and the –308A allele was found during the acute phase (2 weeks) but it did not persist at the end of follow-up or after Bonferroni correction (39). In the second study, SI was associated with the –850 (C/T + T/T) genotype of rs1799724 polymorphism only within the first 2 weeks. The authors also found a significant interaction of –308 G/A and –850 C/T polymorphisms on SI at 1 year after ACS (40). Wang et al. (41) studied three polymorphisms, rs361525, rs1800629, and rs1799964, in a post-mortem sample (SCs and controls). They found no rare allele for rs361525 in the TNFα encoding gene. The allelic and genotypic frequencies of the two other SNPs rs1800629 and rs1799964 were not different between SCs with any psychiatric disorder (including MDD) and healthy control subjects (42).

A Chinese study compared suicidal patients (SAs and/or SI) to non-suicidal patients, all of them affected by schizophrenia. The genotypic and allelic distributions of rs1800629 (–308 G/A) and rs1799964 (–1031 C/T) polymorphisms did not differ between the groups (43).

Lastly, rs1800629 and rs361525 (–238 G/A) polymorphisms have been the subject of one study in patients with schizophrenia with or without a history of SA. The distribution of rs361525 genotype, but not that of rs1800629, was significantly different in SAs compared to individuals with no history of SB (44).

#### TNF-RII

TNF-RII is a protein found mainly on the surface of immune cells that mediates the biological effects of TNF (45). One study on young students after the 2008 Wenchuan earthquake in China investigated, among other things, the role of the *TNF-RII* gene at position + 676 (rs1061622) regarding the susceptibility to SI. Among TT genotype or G allele carriers, women were more likely to report SI than men (46).

#### IL-6

IL-6 is a pro-inflammatory cytokine with a pleiotropic activity on the immune response and inflammation (47). It is produced by T and B cell lymphocytes, macrophages, and glial cells (48) and is one of the cytokines most strongly associated with SIB. Indeed, several post-mortem studies mentioned in meta-analyses and systematic reviews, have shown an abnormally high rate of this cytokine in the blood and brains of SAs or SCs (10, 21, 49).

One study compared the frequencies of two SNPs within the IL-6 encoding-gene, rs2069845 (3329 G/A) and rs1800795 (–174 C/G), between SCs, SAs, and healthy controls (50). The C allele of rs1800795 was significantly more common in SCs than in SAs. Haplotype analysis of these two SNPs revealed that the haplotype AG was more common in SAs compared to healthy controls. The haplotype AC was more common among SCs compared to SAs, and less frequent among SAs compared to controls. The allelic and genotypic frequencies of rs2069845 were not significantly different between the groups.

#### IL-8/CXCL8

Originally known as neutrophil chemotactic factor (NCF), it was later given its current title, IL-8, in 1989 (51). IL-8 plays a role in inflammation (52) and in the development of several cancers (53). Monocytes, neutrophils, fibroblasts, and endothelial cells can release IL-8 (52).

Two studies explored the relationship between IL-8 polymorphisms and SB. In the first of them, female SAs were more likely to present the T allele of rs4073 (–251 A/T) polymorphism than female controls (54). No significant difference was observed among males or in the whole sample. The relationship of this polymorphism with anxiety and depressive symptoms, and with IL-8 plasma levels, was also studied among SAs. The only significant differences concerned anxiety scores, which were lower in subjects with the AA genotype compared with AT and TT.

Another study comparing SCs, SAs and healthy controls found that the T allele of the rs4073 polymorphism was significantly more frequent among SAs than in the other groups (55). The TCA haplotype (rs4073, rs2227306, and rs1126647, respectively) was also more prevalent in SAs compared to SCs. No allelic or genotype differences were found between groups for the rs2227306 and rs1126647 polymorphisms.

#### IL8RA

IL8RA, also known as CXCR1, is the chemokine receptor for the IL-8/CXCL8 (56). This molecule is expressed in a wide variety of cell types: NK cells, mast cells, basophil cells, CD8 + T cells, dendritic cells, and endothelial cells (57). IL8RA plays an essential role in the immune system and inflammation (58, 59). It transfers the signal into the immune cells including neutrophils, lymphocytes, and monocytes.

A follow-up investigation of the Collaborative Study on the Genetics of Alcoholism (COGA) data provided evidence for the involvement of 23 genes, including the IL8RA gene, on alcohol use disorder (AUD) with conduct disorder (CD) and/or SA. Three polymorphisms in this gene (rs16858808, rs16858816, and rs16858811) were associated with the combined phenotype after permutation testing (60).

#### IFNγ

IFNγ is a pro-inflammatory cytokine mainly produced by NK cells, T and B cells, macrophages, and dendritic cells (61). Two studies investigated rs2430561 (+874 A/T) polymorphism and SB. The first one compared allelic and genotypic frequencies of the polymorphism between suicidal subjects (SCs or SAs) and healthy controls. The A/A genotype frequency was significantly higher in males with SB compared to male controls (36). The second study did not find any difference in allelic and genotypic frequencies between MDD subjects with a history of suicide attempts and MDD without such a history (37).

#### IL-18

IL-18, initially called interferon γ inducing factor, is a proinflammatory cytokine that has been implicated in neuroinflammatory and neurodegenerative pathways (62). One study investigated the relationship of two IL-18 promoter polymorphisms, rs187238 (–137 G/C) and rs1946518 (–607 C/A), with suicidal acts and non-suicidal aggression in schizophrenia. The only association concerned rs187238, the frequency of the GC genotype was higher in subjects with schizophrenia and a history of aggression than in healthy controls (63).

#### MCP-1

MCP-1 is a potent inflammatory cytokine. It has a chemoattractant effect on the immune cells. It belongs to the human CC-chemokines family (64, 65). MCP-1 has been associated with several inflammatory disorders like multiple sclerosis (66), rheumatoid polyarthritis (67), and inflammatory bowel disease (68).

One study of Italian outpatients investigated the rs1024611 (–2518 A/G) polymorphism of the MCP-1 encoding-gene in mood disorders (MDD and Type 1 and Type 2 BD. The number of SA was higher in AA genotype carriers compared to AG genotype carriers, independently of diagnosis. The authors showed an association between BD and a history of attempted suicide. Moreover, in the BD group, a higher number of SAs was found in A carriers (69).

#### Migration inhibitory factor

Migration inhibitory factor is a pro-inflammatory cytokine that plays a major role in the regulation of the immune response and inflammation (70). Multiple cell types produce MIF, including T and B cells, monocytes/macrophages, eosinophils, endothelial cells, epithelial cells, fibroblasts, and muscle cells (71). A Japanese study investigated two functional polymorphisms of the *MIF* gene promoter, rs5844572 (794CATT_5–8_ microsatellite) and rs755622 (–173 G/C), in SCs. No significant differences were found in the allelic and genotypic frequency distribution of the rs5844572 and rs755622 polymorphisms between SCs and healthy controls. Haplotype analysis of these polymorphisms also showed no association with suicide (72).

Aytac et al. (73) also investigated rs755622 polymorphism in subjects with BD. The allelic and genotypic frequencies of the SNP were significantly different between SAs, non-SAs and healthy controls (73).

#### IL-10

IL-10 is an immunoregulatory cytokine with anti-inflammatory properties (74). Several cell types synthesize IL-10 such as Th2 cells, monocytes, macrophages, B cells, eosinophils, mast cells and keratinocytes. Three studies examined the relationship between polymorphisms affecting the IL-10 gene and SB. In the first one, the genotype AA of the rs1800896 (–1082 A/G) SNP was more frequent in SCs or SAs compared to healthy controls (36). In the second study, the allele and genotypic frequencies of rs1800871 (–819 C/T) were not statistically different between mental-disordered patients with and without a history of SAs and healthy controls (38). Finally, in the study by Kim et al. (37), the genotypic and allelic distribution of the rs1800896 polymorphism did not differ between two groups of MDD patients: those who had attempted suicide and those who had not (37).

#### IL-1 family

The IL-1 family comprises five members: IL-1α, IL-1β, IL-1ra, IL-18, IL-33 (75), with both pro- and anti-inflammatory properties. IL-1 α, IL-1 β are potent pro-inflammatory cytokines while IL-1ra has anti-inflammatory properties (76). Saiz et al. (35, 77) investigated the relationship between two functional polymorphisms: rs1800587 (IL-1α -889 C/T) and rs1143634 (IL-1β + 3953 C/T) with SB. No significant differences in the genotypic frequency were found between SAs, non-suicidal psychiatric patients, and healthy controls (35, 77). However, the IL-1α -889 TT genotype frequency was higher in SAs that had planned their attempt(s), and the C/T genotype was more common in SAs who made impulsive attempts (77). The aforementioned study by Kang et al. (39) with ACS patients investigated also IL-1β polymorphisms. Two IL-1β alleles, –511T (rs16944) and +3953T, showed a significant association with SI during the acute phase that disappeared after applying Bonferroni correction (39).

#### IL-7

IL-7 is an important cytokine for the development of both B and T lymphocytes [(78), p. 15]. A study conducted on suggestive significant SNPs for SIB reported in GWAS studies found that seven SNPs were significant in different genetic models, particularly, the IL-7 rs10448044 polymorphism in the recessive model (79).

#### TGF-β1

TGF-β1 is a pleiotropic cytokine with pro- and anti-inflammatory activities (80) that is considered a major player in the regulation of the immune response and is produced by many cell types (81). Many studies showed an association between TGF-β1 encoding-gene and inflammatory disorders, asthma (82), systemic sclerosis (83), and rheumatoid polyarthritis (84).

Two studies on SB have been performed on two polymorphisms: rs1800471 (codon 25) and rs1982073 within codon 10, which now bears the number rs1800470 (dbSNP). One study compared depressed SAs and depressed non-attempters to healthy controls, the authors reported the absence of the rare allele in codon 25, but found all three genotypes C/C, C/T, and TT in codon 10. No significant differences in genotypic distribution and allele frequency emerged between the three groups. *In vitro*, TGF-β1 production was higher in depressed patients (suicidal and non-suicidal) compared to healthy controls subjects, but it did not differ between the TGF-β1 genotypes (C/C, C/T, TT) (85). In the second study, SAs were more likely to present the TGF-β1 codon 10 T/T genotype of rs1982073 than healthy controls (86).

#### ACP1

The *ACP1* gene encodes the LMW-PTP (low molecular weight phosphotyrosine protein phosphatase), a tyrosine phosphatase (87) involved in signal transduction pathways required in immune responses (88). This enzyme appears to have a role in the pathophysiology of inflammatory diseases (89).

The rs300774 SNP of ACP1 gene has been associated with SA in a GWAS by Willour et al. (90), and the finding was later replicated by two independent studies. In subjects with BD or MDD with or without SA compared with healthy controls (91), and in males suffering from schizophrenia or schizo-affective disorder with or without history of SA (92). Two different SNPs, rs4447635 and rs7419262, were investigated in a sample of patients with BD and a history of SA. The allelic and genotypic frequencies of rs7419262 were associated with the violence of the suicide attempt (93).

### C-reactive protein

C-reactive protein is an acute-phase inflammatory protein (94). Several SNPs have been identified on the *CRP* gene and the polymorphism rs1130864 (+1444 C/T) has been associated with several diseases. One study compared SAs with various psychiatric diagnoses to healthy controls. An increased risk of SB was found, showing that the T allele was more prevalent among SAs. In addition, the analysis of personality traits in the SA cohort showed that the T allele carriers (CT + TT) had a significantly higher impulsivity score compared with CC carriers (95).

### The kynurenine pathway genes

The kynurenine pathway (KP) is the principal route of tryptophan (TRP) catabolism (96, 97). It is implicated in a wide range of psychiatric (98) and central nervous system disorders (99), but also in SB (100). Several enzymes are involved in the production of KP metabolites, such as quinolinic acid (QUIN) (101, 102), and Picolinic acid (PA) (103). Their products can regulate inflammation and cytokine release (104).

#### Aminocarboxy muconate semialdehyde decarboxylase

The ACMSD enzyme modulates the levels of protective picolinic acid (PA) and toxic quinolinic acid (QUIN). Reduced activity of ACMSD can lead to increased QUIN production. Increased QUIN has been associated with SB (105–107). One study examined the genetic variants of the ACMSD gene related to SB. They showed that the C allele of rs2121337 (T/C) polymorphism was more common in SAs compared to healthy controls (105). The C allele was also associated with increased cerebrospinal fluid levels of QUIN (105). The findings suggest that the rs2121337 polymorphism may influence the activity or expression of ACMSD, and could be linked to SB.

#### Hydroxyanthranilate 3,4-dioxygenase

The HAAO enzyme catalyzes the conversion of 3-HAA acid to acroleyl aminofumarate, which further converts to QUIN and PA. Analysis of the COGA data showed that the HAAO gene SNPs: rs3755541, rs2374442, rs737148, rs3816182, rs13027051, and rs3816184 were significantly associated with the combined AD + CD or SA phenotype after permutation testing (60).

### Genome-wide association studies

Genome-wide association studies have identified novel associations between genetic variants and phenotypes, but they have several limitations: population stratification, missing heritability, epistasis, ultra-rare mutations, and causal variants (108), especially for complex phenotypes like SB.

Although there are few significant associations between SB and SNPs in inflammatory-related genes, some have been reported at the genome-wide significant (GWS) level (*p*-values < 5 × 10^–8^), but some suggestive associations (5 × 10^–8^ ≤ *p*-values < 1 × 10^–5^) also deserve mention.

The rs10903034 SNP, located in the IL28RA gene encodes for a transmembrane protein that heterodimerizes with another subunit to form a type II cytokine receptor (109). This SNP showed a suggestive association with antidepressant-emergent SI [Laje et al., (110)].

Another GWAS study comparing BD subjects with and without a history of SAs, reported an association signal on chromosomal region 2p25 (rs300774) at the threshold of GWS with *p* = 5.07 × 10^–8^ (90). The rs300774 is within a large linkage disequilibrium block containing the acid phosphatase 1 gene (*ACP1*).

The first GWAS study on suicides and non-suicidal deaths found suggestive associations between 22 SNPs of 19 genes and suicide, independently of psychiatric diagnoses. Among these SNPs, the rs1467558 of the CD44 gene is known to be involved in inflammation (111, 112). The CD44 gene was also under-expressed in the SCs group compared to the control group. The same team later performed another GWAS comparing SCs, SAs, psychiatric controls, and healthy volunteers. No GWS SNPs were detected, but two SNPs (rs6480463 and rs4575), within the ADAMTS14 and PSME2 genes which are known to have a regulatory role in inflammatory responses, showed a suggestive association with SB (including SA and SC) (113).

Finally, a GWAS of SA was performed on three BD samples. Two regions showed suggestive associations with SA. One of them was localized to 8q12-q21 (containing rs10448042 and rs10448044), 400 kb upstream of the IL-7 gene (114).

## Discussion

This systematic review found several genetic variants involved in the inflammatory process that could be potentially associated with SIB. Specifically, polymorphisms in IL-8 (rs4073), CRP (rs1130864), TNFα (rs1800629, rs361525, and rs1099724), TNF-RII (rs1061622), TGFβ1 (rs1982073), ACP1 (rs7419262 and rs300774), IL-10 (rs1800896), IFNγ (rs2430561), ACMSD (rs2121337), IL-7 (rs10448044, rs10448042), MIF (rs755622), IL-1α (rs1800587), and IL-1 β (rs1143634 and rs16944). In addition, a GWAS reported one significant association at the threshold of genome-wide significance (GWS) level with the rs300774 SNP, located at the 2p25 region containing the *ACP1* gene. Some suggestive associations were also found with the rs10903034 (IL28RA), rs1467558 (CD44), rs6480463 (ADAMTS14) and rs4575 (PSME2), rs10448042 and rs10448044 (Interleukin-7) polymorphisms.

Five of the SNPs mentioned above have shown a particularly consistent association with the production and/or transcription of inflammatory-related molecules: rs1130864 with CRP, rs1800629 with TNF-α, rs4073 with IL-8, rs1982073 (rs1800470) with TGF-β1, and rs1800896 with IL-10. Below, we briefly describe the changes that have been related to these five polymorphisms:

1) The CRP + 1444T allele (rs1130864) was associated with higher CRP concentrations in a cross-sectional study of 562 European adolescents (115). In another study, homozygosity for the same allele was associated with an increased level of CRP following an inflammatory stimulus (116).
2) Higher TNF-α serum level was found in A carriers (AA/AG) of the rs1800629 polymorphism compared to GG carriers in different clinical samples (117, 118). Also, reporter gene assays demonstrated that the A allele had higher transcriptional activity than the G allele in the human B cell line [Wilson et al. (119)].
3) IL-8 production was significantly higher in A carriers of the rs4073 polymorphism, compared to TT carriers, in blood samples from healthy individuals stimulated with lipopolysaccharide (120). Similarly, in gingival biopsies of chronic periodontitis patients, a higher expression of the IL-8 gene was found in patients with rs4073 AT genotype compared to those with TT genotype (121).
4) Concerning rs1982073, the TGF-β1 serum level was significantly higher in CC/CT carriers than TT carriers in healthy individuals (122) and in peripheral blood mononuclear cells of hypertensive and normotensive individuals, the TGF-β1 mRNA level was higher in CC or TC carriers compared to TT carriers (123).
5) The serum (124) and mRNA level (125) of IL-10 have been associated with the rs1800896.

Overall, the results of this review suggest that genetic variations affecting the inflammatory-related genes may have an influence on the risk of SB. To date, separating the role of inflammatory abnormalities in SB and mood disorders is particularly difficult since the mechanisms overlap and many studies are conducted in mood-disordered samples with no unaffected group (11, 49). However, a recent report based on the NESDA study in the Netherlands suggests the existence of a dose-effect response between inflammation and suicidal risk. This study was based on a large sample of nearly 2000 patients with depressive and/or anxiety disorders and found high levels of CRP and IL-6 in suicide ideators and attempters after controlling for potential confounders and multiple testing (126). A genetic propensity to a more intense inflammatory response might help to identify individuals prone to SIB.

The results should also be considered in light of several limitations. First, we identified a small number of eligible studies. Some studies were flawed by a small sample size. Within the case-control studies that were selected, case samples averaged 260.6 ± 253.5 and ranged from 30 to 1201 samples, while control samples averaged 390.0 ± 352.6 and ranged from 31 to 1479. Testing a single SNP for a relatively uncommon condition (such as SIB) would require 248 cases and ideally 1:4 case-control ratio according to a commonly cited estimation (127). Besides, there is evidence of a publication bias toward positive results in polymorphism studies. Among the studies selected for this review 21 analyzed a low number of polymorphisms (1–3), thus increasing the risk of publication bias. These studies should be considered as providing a low level of evidence (128).

Another selection bias is the fact of using clinical samples with patients affected by diverse psychiatric disorders. This point is important since some of the above-mentioned SNPs have been associated with specific disorders: rs1130864 with anxiety (129), rs1800629 with depression (130, 131), rs2430561 with an increased risk of developing BD (132) and IFN-alpha-induced depression (133).

Also, important confounding factors related to SIB are frequently not considered such as the severity of the suicide attempts or personality traits such as impulsive aggression levels. Low-lethality SAs are phenotypically different from those making violent or severe suicide attempts (15, 134, 135). Most studies included low-lethality attempters (i.e., drug-poisoning) or did not consider this factor (35, 77, 85, 95). The inclusion of high-lethality attempters could yield different results. Another factor for which the analyses are often not adjusted is impulsive aggression, a mediator between genetic vulnerability and the risk of SIB (2, 136, 137). On the other hand, most studies recruit ethnically homogeneous samples or do not control for ethnicity limiting the generalizability of genetic findings.

To date, genetic association studies addressing the involvement of inflammation-related genes in SIB have been performed without considering potential interactions between these SNPs and other factors, such as gene-gene (GxG), gene-environment (GxE), and epigenetic interactions. This severely limits the understanding of genetic mechanisms in SIB. For instance, in patients with schizophrenia, a GxG interaction between the rs16940665 of the corticotrophin-releasing hormone receptor type 1 (CRHR1) and the rs1875999 in CRH binding protein increases the risk of SB (138). One GxE study found that the interaction between childhood trauma and FK506-binding protein 5 (FKBP5) SNPs and haplotypes may influence SB (139, 140). Epigenetic mechanisms, such as DNA methylation (141), microRNAs (41), and histone modifications (142) could also increase the risk of SB.

In summary, several SNPs that could affect the production or expression of inflammatory-related molecules and modify the intensity of the inflammatory response have been associated with SIB. However, the extant studies presenting noteworthy limitations, more comprehensive and larger studies, as well as novel approaches based on GxG or GxE, are warranted.

## Data availability statement

The original contributions presented in this study are included in the article/Supplementary material, further inquiries can be directed to the corresponding author.

## Author contributions

RT and JL-C conceptualized the study. RT conducted the literature search and drafted the manuscript. All authors reviewed the manuscript and approved the submitted version.
