# Supplementary material for: Genetic variations related to inflammation in suicidal ideation and behavior: A systematic review
Source: Front Psychiatry. 2022 Oct 17;13:1003034. doi: 10.3389/fpsyt.2022.1003034 (PMC9621324; doi:10.3389/fpsyt.2022.1003034)
Supplement: Supplementary file 1 [file Data_Sheet_1.docx]

**Annex**

**Table S1.** Quality assessment of included studies using the ‘Q-Genie’ quality assessment tool.

| **Study** | **Rationale for study** | **Selection and definition of outcome of interest** | **Selection and comparability of comparison groups** | **Technical classification of the exposure** | **Non-technical classification of the exposure** | **Other sources of bias** | **Sample size and power** | **A priori planning of analyses** | **Statistical methods and control for confounding** | **Testing of assumptions and inferences for genetic analyses** | **Appropriateness of inferences drawn from results** | **Score** | **Quality level** |
| --- | --- | --- | --- | --- | --- | --- | --- | --- | --- | --- | --- | --- | --- |
| Saiz et *al*., 2008 | 4 | 5 | 4 | 2 | 2 | 2 | 3 | 3 | 2 | 2 | 3 | 32 | Poor |
| Laje et *al*., 2009 | 4 | 5 | 3 | 4 | 2 | 2 | 2 | 4 | 4 | 3 | 3 | 36 | Moderate |
| Omrani et *al*., 2009 | 3 | 3 | 2 | 2 | 2 | 1 | 2 | 2 | 1 | 2 | 2 | 22 | Poor |
| Altamura et *al*., 2010 | 7 | 6 | 5 | 2 | 2 | 3 | 2 | 3 | 2 | 4 | 4 | 40 | Moderate |
| Lee and Kim., 2010 | 5 | 5 | 4 | 2 | 2 | 3 | 2 | 4 | 3 | 3 | 3 | 36 | Moderate |
| Dick et *al*., 2010 | 7 | 6 | 4 | 2 | 1 | 2 | 3 | 3 | 3 | 3 | 3 | 37 | Moderate |
| Saiz et *al*. 2011 | 5 | 5 | 5 | 2 | 3 | 2 | 3 | 5 | 2 | 3 | 4 | 39 | Moderate |
| Liu et al., 2011 | 6 | 5 | 3 | 4 | 2 | 2 | 4 | 3 | 2 | 2 | 3 | 36 | Moderate |
| Willour et *al*., 2012 | 7 | 7 | 7 | 5 | 4 | 4 | 4 | 4 | 2 | 5 | 5 | 54 | Good |
| Omrani et *al*,. 2012 | 3 | 3 | 3 | 1 | 1 | 1 | 1 | 1 | 1 | 2 | 2 | 19 | Poor |
| Kim et *al*., 2013 | 7 | 7 | 4 | 1 | 1 | 4 | 3 | 6 | 5 | 4 | 4 | 46 | Good |
| Suchankova et *al*., 2013 | 3 | 1 | 2 | 2 | 1 | 1 | 2 | 2 | 2 | 2 | 2 | 20 | Poor |
| Galfalvy et *al*., 2013 | 7 | 6 | 3 | 2 | 2 | 3 | 3 | 3 | 3 | 3 | 3 | 38 | Moderate |
| Janelidze et al., 2015 | 7 | 7 | 7 | 4 | 1 | 2 | 3 | 6 | 1 | 4 | 4 | 46 | Good |
| Zai et *al*., 2015 | 7 | 6 | / | 5 | 1 | 1 | 2 | 3 | 2 | 3 | 3 | 33 | Moderate |
| Galfalvy et *al*., 2015 | 4 | 4 | 4 | 3 | 1 | 2 | 5 | 5 | 3 | 4 | 4 | 39 | Moderate |
| Brundin et al., 2016 | 7 | 6 | 4 | 4 | 2 | 3 | 4 | 5 | 4 | 4 | 4 | 47 | Good |
| Pawlack., et *al* 2016 | 6 | 4 | 3 | 3 | 3 | 3 | 4 | 4 | 5 | 4 | 4 | 43 | Moderate |
| de Medeiros Alves et al., 2017 | 7 | 7 | 3 | 4 | 1 | 2 | 2 | 6 | 5 | 5 | 5 | 47 | Good |
| Campos., et *al* 2017 | 5 | 3 | 3 | 3 | 3 | 3 | 4 | 3 | 2 | 2 | 2 | 33 | Poor |
| Shimmyo et *al*., 2017 | 3 | 2 | 3 | 2 | 2 | 2 | 2 | 2 | 2 | 3 | 2 | 25 | Poor |
| Li et *al*., 2017 | 6 | 4 | 3 | 3 | 3 | 3 | 4 | 5 | 3 | 3 | 4 | 41 | Moderate |
| Kang., Et *al* 2017 | 5 | 4 | 3 | 3 | 3 | 3 | 4 | 4 | 4 | 3 | 3 | 39 | Moderate |
| Wang et *al*., 2018 | 3 | 3 | 3 | 3 | 3 | 3 | 2 | 3 | 2 | 3 | 3 | 31 | Poor |
| Noroozi et *al*., 2018 | 7 | 6 | 3 | 2 | 1 | 2 | 3 | 3 | 2 | 4 | 4 | 37 | Moderate |
| Eftekharian et al., 2018 | 7 | 7 | 4 | 3 | 2 | 4 | 3 | 4 | 4 | 4 | 4 | 46 | Good |
| Gupta et *al*., 2020 | 5 | 3 | 3 | 2 | 2 | 2 | 2 | 2 | 2 | 2 | 2 | 27 | Poor |
| Lang et *al*., 2020 | 6 | 6 | 5 | 4 | 4 | 2 | 2 | 3 | 3 | 2 | 4 | 41 | Moderate |
| Aytac et *al*., 2020 | 4 | 4 | 2 | 1 | 1 | 2 | 1 | 3 | 2 | 2 | 2 | 24 | Poor |
| Aytac et *al*., 2022 | 4 | 4 | 3 | 2 | 2 | 2 | 2 | 2 | 3 | 2 | 2 | 28 | Poor |
| Kang et *al*., 2021 | 6 | 4 | 3 | 3 | 3 | 3 | 4 | 4 | 4 | 3 | 3 | 40 | Moderate |
| Aytac et *al*., 2022 | 5 | 4 | 4 | 2 | 3 | 3 | 3 | 3 | 3 | 3 | 3 | 36 | Moderate |

The Q-Genie Tool containe 11 items or question rated on a scale of 1 to 7 assessing the domains above. Following evaluation, an overall score can indicate studies of poor (Scores ≤35), moderate (>35 and ≤45) or good quality (>45). For studies without control groups: poor quality (Scores ≤32), moderate quality (>32 and ≤40), good (>40).

**List of abbreviations**

ACMSD: Aminocarboxymuconate Semialdehyde Decarboxylase

ACP1: Acid phosphatase 1

AD: Alcohol Dependence

ADAMTS14: A Disintegrin And Metalloproteinase with Thrombospondin Motifs 14

AUD: Alcohol Use Disorder

BD: Bipolar Disorder

CD: Conduct Disorder

CD44: [Cluster of differentiation](https://www-sciencedirect-com.ezpum.biu-montpellier.fr/topics/immunology-and-microbiology/cluster-of-differentiation)

COGA: Collaborative Study on the Genetics of Alcoholism

CRHR1: Corticotrophin-Releasing Hormone Receptor Type 1

CRP: C-reactive protein

CXCL: Chemokine (C-X-C motif) Ligand

FKBP5: FK506-binding protein 5

GWAS: Genome-Wide Association *Study*

GWS: Genome-Wide Significant

HAAO: Hydroxyanthranilate 3,4-Dioxygenase

IFNγ: interferon Gamma

IL-10: Interleukin 10

IL-18: Interleukin 18

IL-1α: Interleukin 1 alpha

IL-1β: Interleukin 1 beta

IL-2: Interleukin 2

IL-2R: Soluble Interleukin 2 Receptors

IL-4: Interleukin 4

IL-6: Interleukin 6

IL-7: Interleukin 7

IL-8: Interleukin 8

IL28RA: Interleukin 28 Receptor Alpha

IL8RA: Interleukin 8 Receptor Alpha

KP: Kynurenine Pathway

LMW-PTP: Low Molecular Weight Phosphotyrosine protein Phosphatase

MCP-1: Monocyte chemoattractant protein 1

MDD: Major Depressive Disorder

MeSH: Medical Subject Headings

MIF: Macrophage migration inhibitory factor

mRNA: Messenger Ribonucleic Acid

NLR: Neutrophil‐To‐Lymphocyte Ratio

PA: Picolinic acid

PSME2: proteasome activator complex subunit 2

QUIN: Quinolinic Acid

SA: Suicide Attempter

SB: Suicidal Behavior

SC: Suicide Completer

SD: Standard deviation

SI: Suicidal Ideation

SIB: Suicidal Ideation and Behavior

SNPs: Single Nucleotide Polymorphisms

TGF-β1: Transforming Growth Factor Beta

TNF-RII: Tumor Necrosis Factor Receptor 2

TNFα: Tumor Necrosis Factor Alpha

TRP: Tryptophan

**Search terms used:**

**PubMed**

("Suicide"[Title/Abstract] OR "suicidal ideation*"[Title/Abstract] OR "suicidal behavior"[Title/Abstract] OR "suicidal thought*"[Title/Abstract] OR "suicide attempt*"[Title/Abstract] OR "suicidality"[Title/Abstract] OR "Suicide"[MeSH Terms]) AND ("inflammation"[Title/Abstract] OR "inflammatory marker*"[Title/Abstract] OR "inflammation response*"[Title/Abstract] OR "immune system"[Title/Abstract] OR "immune regulation"[Title/Abstract] OR "immune system deregulation"[Title/Abstract] OR "inflammatory"[Title/Abstract] OR "cytokines"[Title/Abstract] OR "interleukines"[Title/Abstract] OR "chemokines"[Title/Abstract] OR ("inflammation"[MeSH Terms] OR "cytokines"[MeSH Terms])) AND ("genetics"[Title/Abstract] OR "genetic variation*"[Title/Abstract] OR "single nucleotide polymorphism*"[Title/Abstract] OR "SNP"[Title/Abstract] OR "epigenetic*"[Title/Abstract] OR "gene expression regulation"[Title/Abstract] OR "gene expression"[Title/Abstract] OR ("genetics"[MeSH Terms] OR "genetic variation"[MeSH Terms] OR "gene expression regulation"[MeSH Terms]))

**PsychoINFO**

Suicide or suicidal ideation or suicidal behavior or suicidal thoughts or suicide attempt or suicidality

AND

Inflammation or inflammatory or inflammatory marker or inflammation response or immune system or immune regulation or immune system deregulation

AND

Genetics Genetics or genetic variation or single nucleotide polymorphism* or SNP [TIAB] or epigenetic* or [Gene Expression Regulation](https://www.ncbi.nlm.nih.gov/mesh/68005786) or gene expression
